# The NICE search filters for treating and managing COVID-19: validation in MEDLINE and Embase (Ovid)

**DOI:** 10.5195/jmla.2024.1806

**Published:** 2024-07-01

**Authors:** Paul Levay, Amy Finnegan

**Affiliations:** 1 paul.levay@nice.org.uk Senior Information Specialist, National Institute for Health and Care Excellence, Manchester, United Kingdom; 2 amy.finnegan@nice.org.uk Senior Information Specialist, National Institute for Health and Care Excellence, Manchester, United Kingdom

**Keywords:** Search filters, COVID-19, MEDLINE, Embase, Systematic literature review

## Abstract

**Objective:**

In this paper we report how the United Kingdom's National Institute for Health and Care Excellence (NICE) search filters for treating and managing COVID-19 were validated for use in MEDLINE (Ovid) and Embase (Ovid). The objective was to achieve at least 98.9% for recall and 64% for precision.

**Methods:**

We did two tests of recall to finalize the draft search filters. We updated the data from an earlier peer-reviewed publication for the first recall test. For the second test, we collated a set of systematic reviews from Epistemonikos COVID-19 L.OVE and extracted their primary studies. We calculated precision by screening all the results retrieved by the draft search filters from a targeted sample covering 2020–23. We developed a gold-standard set to validate the search filter by using all articles available from the “Treatment and Management” subject filter in the Cochrane COVID-19 Study Register.

**Results:**

In the first recall test, both filters had 99.5% recall. In the second test, recall was 99.7% and 99.8% in MEDLINE and Embase respectively. Precision was 91.1% in a deduplicated sample of records. In validation, we found the MEDLINE filter had recall of 99.86% of the 14,625 records in the gold-standard set. The Embase filter had 99.88% recall of 19,371 records.

**Conclusion:**

We have validated search filters to identify records on treating and managing COVID-19. The filters may require subsequent updates, if new SARS-CoV-2 variants of concern or interest are discussed in future literature.

## BACKGROUND

Reliable and effective literature searches are required for research topics about COVID-19 and SARS-CoV-2. This paper presents validated search filters that can be applied in literature search strategies to identify evidence on treating and managing COVID-19. There is an ongoing need to undertake literature searches on COVID-19, even now that the public health emergency has ended. COVID-19 remains a global health threat leading to death, hospitalization and significant consumption of healthcare resources [1]. It is important to have effective search filters to help us deal with the high volume of research that has characterized the pandemic [2].

Search filters are sets of validated search terms that retrieve records with a common feature from bibliographic databases [3]. Search filters aim to maximize the retrieval of records sharing this common feature (recall) and to minimize the retrieval of records that do not share it (precision). Filters are tested using a gold-standard set of records known to contain that common feature [4]. One method of creating a gold-standard set is hand searching to identify relevant papers that the filter should retrieve. An efficient alternative approach is relative recall, which involves pooling papers found during previous searches that are known to represent the common feature of interest to the filter [5].

The filters we present here have been developed for the MEDLINE and Embase databases using the Ovid platform [6, 7]. We expect these search filters will be used in combination with search terms to describe the management and treatment interventions of interest, such as drugs, devices, surgical procedures and other therapeutics.

### Purpose of the Paper

The search filters originate in the work we did to support the National Institute for Health and Care Excellence (NICE) in developing rapid evidence-based guidelines for the United Kingdom (UK). The draft search filters tested in this paper were taken from the most recent versions in use at NICE. The development process, showing how the filters evolved, is summarized in Appendix A.

NICE uses the best available evidence to develop recommendations on a range of health and social care topics [8]. In March and April 2020, NICE produced 21 rapid guidelines on identifying symptoms and complications of COVID-19, therapeutic interventions, protecting people with clinically vulnerable conditions and managing health services [9]. The rapid guidelines were maintained using weekly surveillance searches until April 2023. The search strategies were developed specifically for the NICE remit of treating and managing COVID-19. The strategies also required maintenance throughout that period.

The purpose of this paper is to report on how we finalized the draft strategies and validated them as search filters. In June 2021, we published a preprint with a detailed description of the development process [10]. We intended the preprint to be an interim publication to meet an urgent need during a public health emergency, as a way of encouraging information specialists to collaborate [11]. We did not feel it was appropriate to do validation while new terminology and concepts relating to COVID-19 were still emerging. Since then, the information landscape has changed, and it is appropriate to undertake this validation.

### Developing the Search Strategies for NICE

We created version 1 of the search strategies on March 16, 2020, and developed them iteratively during the subsequent weeks to support the rapid guidelines. There had not been any agreed terminology until February 2020 when the World Health Organization (WHO) named the condition “COVID-19” and the virus causing it “SARSCoV-2” [12]. It took time for the WHO naming conventions to be used in the literature and we needed to account for new and changing terminology during this period of the pandemic. We adopted the concept of the “living search strategy” and kept the search terms we were using under continual review [13].

We kept the search strategies up to date with regular testing. We made modifications in spring 2021, when Ovid updated the Medical Subject Headings (MeSH) available in MEDLINE and the Emtree thesaurus in Embase. Adding the new subject headings for COVID-19 and SARS-CoV-2 meant we could rationalize the free-text terms we used in the search strategies (see Appendix B for terms we have not included in the final filters). The objective testing we carried out for each free-text term was fully reported in the preprint [10]. Our testing showed that we would not miss any records relevant to NICE, while the improved precision meant we would have fewer irrelevant records to review, as we kept the rapid guidelines up to date. We published these results as version 10 in the preprint in June 2021 [10].

In April 2023, we created version 12 by adding free-text terms and subject headings to retrieve records relating to the Omicron variant of SARS-CoV-2 [14]. We were retrieving records on Delta and other Variants of Interest (VOI) or Variants of Concern (VOC) without needing to make further modifications. Details on how we accounted for earlier variants are available in Appendix A and Appendix B.

We have used version 12 of the search strategies as the draft search filters in this paper. We have not reported the three-year development period as that has been covered elsewhere [10]. In Appendix C to this paper we have provided a list and description of the online-only supporting materials that we have made available through Open Science Framework (OSF). These supporting files provide the data and search strategies we used in testing and validating the filters. As listed in Appendix C, online-only supporting File A in OSF provides the full search strategies for each version of the filters.

### Alternative COVID-19 Search Approaches

We are not aware of any other validated search filters on COVID-19 or SARS-CoV-2. No validated search filters were listed on the Information Specialists' Subgroup (ISSG) Search Filter Resource on December 18, 2023 [15].

We are aware of several search strategies designed for PubMed, eight of which were tested prior to May 2020 [16]. The most sensitive strategy had a recall of 98.7%, although it would need to be adapted to the Ovid platform [13]. It is unclear how changes in terminology will have affected performance of these strategies.

Study-based registers became an important way to access evidence on COVID-19. These registers are usually open access, collating records from several sources to give users a single point of entry to the literature [17]. Reviews of COVID-19 study-based registers, including the Cochrane COVID-19 Study Register [18] and Epistemonikos COVID-19 L.OVE [19], have found them to be sufficiently comprehensive and up to date for use in systematic reviews [20–22]. The Study Classifier used to maintain the Cochrane COVID-19 Study Register had recall of 98.9% and precision of 63.8% [22]. These evaluations of study-based registers assessed their overall coverage and not the effectiveness of the individual search strategies they use on the various databases.

While we found study-based registers useful for the rapid NICE guidelines, it was still necessary to use our own search strategies. The functionality of the registers meant that they could not wholly replace separate searches of each database. For example, we were running weekly searches throughout 2021–2023 for 100 pharmaceutical products for NICE, which required a saved search strategy with over 200 free-text terms, application of date limits, large exports of data and other features not available from the study-based registers. There is still a need for validated COVID-19 search filters for MEDLINE and Embase.

### Aim and Objectives

The aim was to validate search filters to retrieve records from the Ovid versions of MEDLINE and Embase that are optimized for use in searches on treating and managing COVID-19.

The targets were 98.9% for recall and 64% for precision, to at least match the Cochrane COVID-19 Study Register [22].

The objectives were to:

- Test the draft search filters in MEDLINE and Embase and make any modifications.
- Collate a gold-standard set of records relevant to treating and managing COVID-19.
- Validate the draft search filters and calculate relative recall.
- Create an appropriate sample and use it to calculate the precision of the search filters.

## METHODS

### Definitions

**Figure 1:**
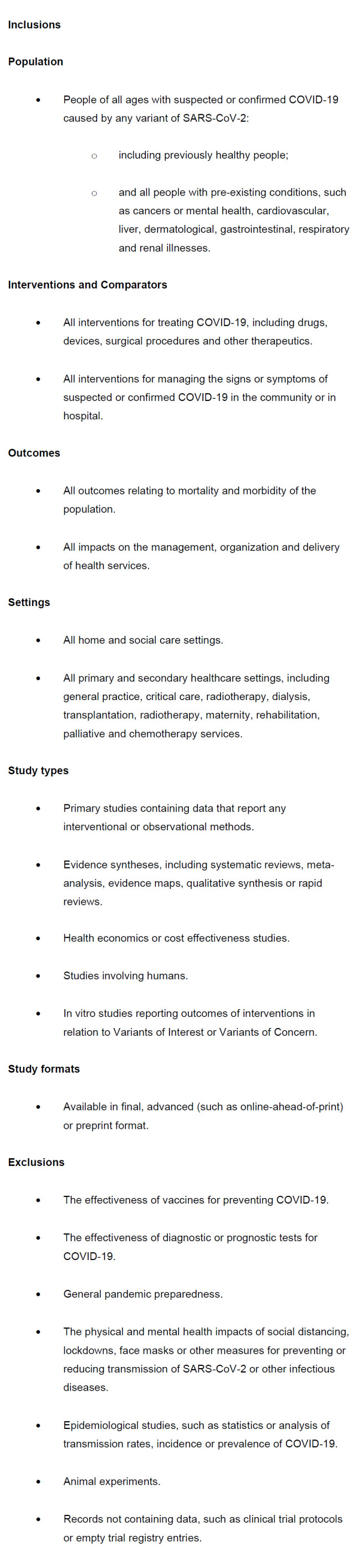
Definitions used when testing and validating the search filters

We used the definitions in Figure 1 to set both the parameters of the filters and to make the screening decisions during testing. We used “relevance” in this context to mean a record that should be retrieved by a search (recall testing) or should not be retrieved (precision testing) for further assessment. We did not judge relevance according to whether the full text of a paper would be includable in a NICE rapid guideline.

The purpose of the filters is to retrieve records from the Ovid versions of MEDLINE and Embase about treating and managing COVID-19 in people of all ages in the community or in hospital. The filters are not optimized for retrieving records about diagnosis, prognosis, transmission, prevention, vaccination, mechanisms of action, epidemiology, or etiology. The filters are not validated for diagnosing, managing or treating secondary conditions caused by COVID-19, including long covid or post-COVID-19 syndrome.

### Testing to Finalize the Draft COVID-19 Search Filters

We undertook four tests to finalize and validate the draft COVID-19 search filters: two to check recall, one for precision and one to test the relative recall of the gold-standard set.

Recall, also known as sensitivity, is:

- the proportion of available, relevant results that a search filter retrieves.
- calculated as the number of relevant records retrieved, divided by the total number of relevant records in the test set (expressed as a percentage).

Precision is:

- the proportion of records retrieved by a search filter that are relevant.
- calculated as the number of relevant records retrieved, divided by the total number of records retrieved (expressed as a percentage) [23].

We recorded all the screening decisions in EPPI-Reviewer version 5 (EPPI-R5). We undertook the MEDLINE tests in MEDLINE ALL, which is the Ovid-recommended method to access MEDLINE, Epub Ahead of Print, In Process&In Data Review Citations, and the other segments [7]. We carried out the Embase tests in the segment with a start date of 1974 [6].

#### Recall Test 1: Set Obtained from Butcher et al.

The first recall test used the set collated for a published article assessing the completeness of COVID-19 study-based registers [24]. Butcher et al. had identified systematic reviews meeting their criteria from Epistemonikos COVID-19 L.OVE, from which they extracted primary studies. We chose this test set as it was collated by a separate, independent research team and their methods had already been peer reviewed. The methods they used to collate their test set have been fully reported [24].

We received an Excel spreadsheet from the lead author of the study listing their test set (see File E in our online-only supporting materials posted to OSF). We cleaned the data for use in our own test and removed duplicates. We removed any grey literature reports that were not indexed on MEDLINE or Embase. We checked the preprints listed in the test set to see if a later, peer-reviewed, article had been published. We did this by checking the preprint on medRxiv or bioRxiv for links to a later article, then, where these did not exist, we searched for title words and authors in Ovid. When we identified later articles, we added these alongside the original preprints in order to update the test set. We created a new search strategy in Ovid for the test set using the Digital Object Identifier (DOI) where already known or the title. We combined the draft COVID-19 search filters with the test set in Ovid and recorded which items were retrieved (see OSF supporting Files B and C for details).

#### Recall Test 2: Updated Supplementary Sets

We used a second recall test to assess the draft search filters with a more up-to-date set of papers. As the test set from Butcher et al. had been collated in late 2020, it did not cover the variants of interest or concern that emerged afterwards. We followed a similar process to be consistent with the first test. We applied the category “Prevention and Treatment” in Epistemonikos L.OVE to identify relevant systematic reviews on COVID-19. We searched within these, using the title, abstract, author, and journal fields for the terms: “delta” or “variants of concern” or “variant of concern” or “variants of interest” or “variant of interest” or “omicron.”

We screened the remaining records according to our criteria in Figure 1 to remove the prevention and epidemiology reviews.

We used citationchaser to identify the reference lists from the reviews we selected from our Epistemonikos search. Citationchaser is a free and open-source Shiny app that uses data from The Lens.org to conduct citation searching [25]. We extracted the DOI for each review and pasted the list into citationchaser. We downloaded the reference lists from citationchaser as RIS files, uploaded them to EPPIR5, removed duplicates and screened the results according to the definitions in Figure 1. Both authors (who had been making decisions on relevance for these strategies for three years at this point) did the screening independently. We reconciled any discrepancies through discussion.

We had two new test sets, comprising the systematic reviews from Epistemonikos and the relevant primary studies obtained from their reference lists. We identified these items in Ovid using the DOI field, which we extracted from the citationchaser records (see OSF supporting File F). We combined the draft search filters with these test sets in Ovid and recorded which items were retrieved.

#### Precision Test

As the draft search filters would retrieve over half a million results from each database, we needed to download a sample to ensure we could feasibly complete the screening with the time and resources available. We needed a sample that would reflect the changing terminology from 2020 to 2023. There were also long periods when each variant of interest or concern would not have been referred to in the literature. Given the need to account for these factors, a targeted sample was more useful than a random sample.

We decided to download all the results from our draft search filters that had been added to MEDLINE and Embase on a single calendar day. We could then download all records from that day in 2020, 2021, 2022 and 2023, giving us a sample from throughout the pandemic. As Ovid only adds records to the databases from Monday to Friday, the date chosen needed to have been a working day in each of the four years. The day needed to be after February 22nd, to account for when WHO named COVID-19 in 2020. It also needed to be a day that had already passed in 2023 so that records would be available for the test.

We ran the draft search filters in Ovid and limited them to the relevant four dates. We used the fields Create Date (.dt) and Entry Date (.ed) in MEDLINE and Date Created (.dc) in Embase to generate the sample. We dual screened the records for relevance to COVID-19 and SARS-CoV-2 in EPPI-R5 (see OSF supporting Files G and H). We deduplicated the results to calculate a combined precision figure, as, in practice, both databases would normally be used in a literature search.

**Figure 2:**
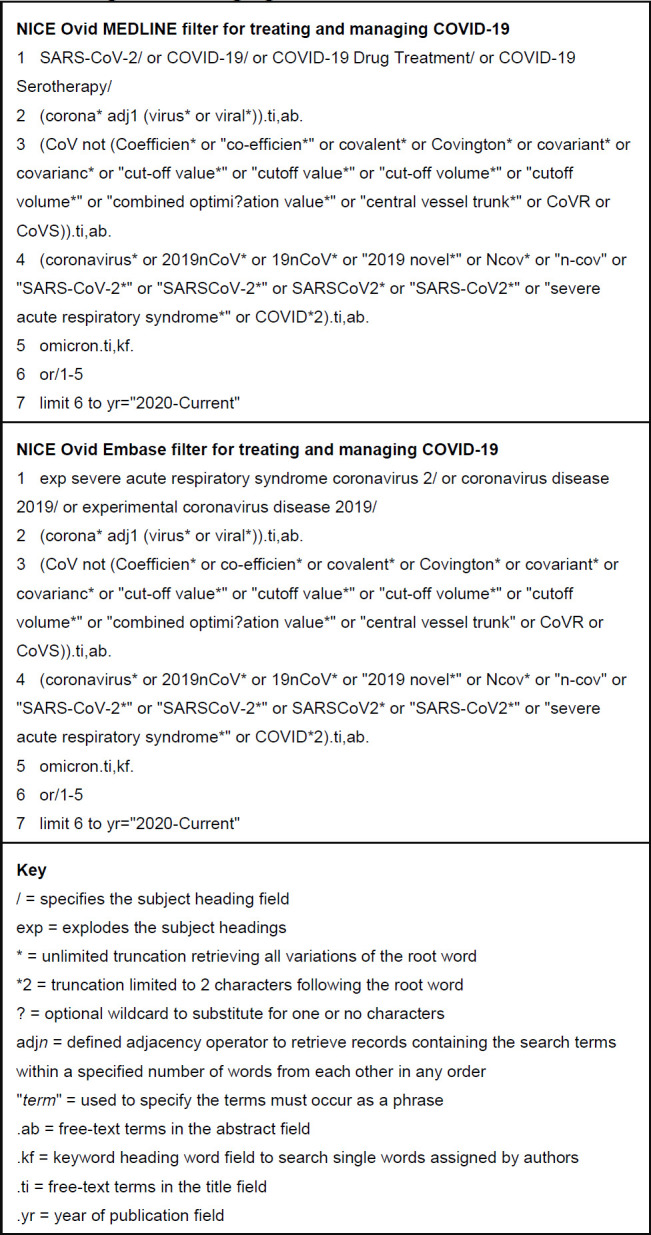
The NICE MEDLINE and Embase (Ovid) search filters for treating and managing COVID-19.

We collated the records that we had marked as not relevant from the deduplicated set (see OSF supporting File I). We added the titles and abstracts of these records to the word and phrase counters freely available at <www.rapidtables.com>. We ran the counters to identify whether the irrelevant records contained any frequently occurring single words or two-word or three-word phrases (see OSF supporting File J). We assessed whether the words or phrases frequently appearing in the irrelevant records could be excluded from the draft search filters to make them more precise (as demonstrated in Figure 2, where we use the NOT operator to make Line 3 more precise).

#### Validation

We validated the search filters by testing the relative recall of a gold-standard set of records that we had not previously seen. We had used internal NICE data from the rapid guidelines to develop the draft search filters, therefore, we needed to collate a new gold-standard set to prevent biased results.

We created the gold-standard set by using the Cochrane COVID-19 Study Register [18]; a source we knew contained reliable and comprehensive evidence on COVID-19 [20, 22]. Cochrane used a range of sources rather than a single search strategy to collate the Study Register, which meant we would obtain a set that could be used in both MEDLINE and Embase. We knew that Cochrane had assessed the relevance of the studies to COVID-19 and so they would be appropriate for our gold-standard set [22]. This method meant we could create a much larger set than if we hand searched for relevant records [5].

We applied the “Treatment and Management” subject filter and the “Journal Article” study-type filter in the Cochrane COVID-19 Study Register. We downloaded all the results into CSV files. The export limit meant we had to do this in batches, with results limited by year of creation. We collated a master list from the CSV files in Excel and cleaned the data. We removed the clinical trial registry records, so that we retained articles and preprints. We extracted identifying numbers from Excel, including PubMed ID (.ui), DOI (.do) or Embase Accession Number (.an) and searched for these in Ovid. Where no number was available, we searched by title. We used these methods to ensure our Embase gold-standard set covered the records that Cochrane had obtained from MEDLINE or other sources.

We ran searches for the gold-standard set in Ovid, downloaded RIS files and imported them to EPPI-R5, where we removed any duplicates. We did further data cleansing to remove obviously irrelevant records, such as where numerous records were retrieved because the same DOI was applied to all conference papers published in a single journal supplement (ensuring we retained the one record of relevance).

We exported the PubMed ID field from EPPI-R5 in batches of 1000. We converted these lists of ID numbers into Ovid search strategies, which we pasted into MEDLINE. We ran our draft COVID-19 search filter. We used the search format “Gold standard AND Draft search filter” in Ovid to test recall and we used “Gold standard NOT Draft search filter” to identify any records we would miss. We followed the same process with Embase, having collated the accession numbers for the gold-standard set into an Ovid search strategy. We collated the records missed by the filters and tabulated their characteristics. The search strategies for the validation tests are available in supporting Files B and C, while the gold-standard sets are available in supporting Files K and L on OSF.

## RESULTS

### Testing to Finalize the Draft COVID-19 Search Filters

#### Recall Test 1: Set Obtained from Butcher et al.

The lead author sent us a list of 440 records that had been used for the completeness test in their original article [24]. Four of these records were grey literature reports that we could not identify in MEDLINE or Embase on April 17, 2023. We removed three duplicates from the list. We identified that 30 of the 440 records were preprints and established that 16 of these had later articles associated with them. Our final test set comprised 449 records.

We ran the tests on April 24, 2023, using the Ovid segments dated April 21, 2023. Our draft search filters retrieved 409 of the 411 records available on MEDLINE and 392 of the 394 available on Embase, giving us a recall rate of 99.5% in both databases (see Table 1).

**Table 1:** Performance of the draft search filters in recall test 1 with the test set obtained from Butcher et al. (April 24, 2023).

Test set	MEDLINE	Embase
No. in test set	449	449
No. available on database	411	394
No. retrieved by filter	409	392
Percentage of those available retrieved by filter	99.5%	99.5%

The draft search filters missed the same two records in both databases (see OSF supporting File E). We examined the free text and subject headings of these records. One paper was about endocarditis [26] and the other was about Gitelman syndrome [27]. We decided that these papers did not meet our screening criteria in Figure 1, despite being in the COVID-19 test set from Butcher et al. [24]. We did not alter our draft search filters, as we had already exceeded our recall target of 98.9%.

#### Recall Test 2: Updated Supplementary Sets

On April 19, 2023, Epistemonikos L.OVE contained 15,056 systematic reviews and 7679 of these were tagged with “Prevention or Treatment”. We searched within these for the terms relating to variants listed above and found 116 results. We screened the 116 records and included 33 and excluded 83 of these systematic reviews. We found that 30 of the 33 reviews were available on citationchaser and that these had a combined total of 1484 records in their reference lists. We downloaded a RIS file containing the papers in these references lists. In EPPI-R5 we removed 41 duplicates and dual screened the remaining 1443 records according to the criteria in Figure 1. From this screening, we identified 1049 records that we could use in the supplementary test set of primary studies (see OSF supporting File F).

We ran the test on April 25, 2023, using the Ovid segments dated April 24, 2023. We found our draft search filters had 100% recall of the systematic reviews, with 27 available on MEDLINE and all 33 available on Embase (see Table 2). We ran the primary studies test and the draft search filters retrieved 924 of the 927 available on MEDLINE (99.7%) and 949 of the 951 available on Embase (99.8%).

**Table 2:** Performance of the draft search filters in recall test 2 with the updated supplementary sets (April 25, 2023).

Category	Test set	MEDLINE	Embase
Systematic reviews	No. in test set	33	33
	No. available on database	27	33
	No. retrieved by filter	27	33
	Percentage of those available retrieved by filter	100%	100%
Primary studies	No. in test set	1049	1049
	No. available on database	927	951
	No. retrieved by filter	924	949
	Percentage of those available retrieved by filter	99.7%	99.8%
To tell test set	No. in test set	1082	1082
	No. available on database	954	984
	No. retrieved by filter	951	982
	Percentage of those available retrieved by filter	99.7%	99.8%

We examined the four different records we missed: there were three in MEDLINE [28–30] and two in Embase [29,31] (see also OSF supporting File F). We found that none of the four had abstracts, only one had subject headings and three were letters. It was not possible to retrieve these records without adversely affecting precision. For example, two could only be retrieved by searching for the drug name “molnupiravir” [29,31] (at a time NICE was monitoring over 100 pharmaceutical products). Again, we had exceeded our recall target of 98.9% and so we moved to our next test without making further changes to the draft search filters.

#### Precision Test

We chose the date of April 28 for the precision test as it had been a working day each year from 2020–2023. We ran the test on May 4, 2023, using the Ovid segments for May 3, 2023. The draft search filter had 354,166 results in MEDLINE, of which 2633 had been added on April 28 in 2020–2023. The draft Embase filter had 454,578 results and we downloaded the 712 that had been added on April 28 each year. We verified that records were added to both databases for each year of the test period. We uploaded the samples to EPPI-R5 for screening. We created a combined file, from which we removed 72 duplicates, to leave 3273 records for the overall test of precision (see Table 3).

**Table 3:** Performance of the draft search filters in the precision test (May 4, 2023).

Screening decision	No. downloaded from MEDLINE		No. downloaded from Embase		Total after deduplication	
	Number	Percentage	Number	Percentage	Number	Percentage
Include: Relevant to COVID-19 or SARS-CoV-2	2402	91.2%	643	90.3%	2982	91.1%
Exclude: Relevant to other coronaviruses	24	0.9%	2	0.3%	26	0.8%
Exclude: Not relevant	207	7.9%	67	9.4%	265	8.1%
Total in test set	2633	100%	712	100%	3273	100%

We found that the draft search filters had a precision of 91.2% in MEDLINE and 90.3% in Embase (see Table 3). In the overall test of the deduplicated sample, we found that 2982 records (91.1%) were relevant and 291 (8.9%) were not relevant (see Table 3). The 291 irrelevant records included 26 (0.8% of the total) that were about other coronaviruses (such as Middle East Respiratory Syndrome (MERS)) but not COVID-19. We had found during development that we needed to include the free-text term “coronavirus” (as it is part of the name “Coronavirus Disease 2019”) and we did not want to harm recall by removing it.

The other 265 irrelevant records (8.1% of the total) in the deduplicated sample included a number of papers that referred to the COVID-19 pandemic, although they were not relevant to our criteria in Figure 1. For example, we excluded a review of how students adapted to online learning during COVID-19 lockdowns.

We added the titles and abstracts of the 291 excluded articles to <www.rapidtables.com> on May 17, 2023, and sorted the resulting words and phrases according to the number of occurrences. After eliminating terms referring to study types, such as “scoping review” and “case study”, the most frequent two-word phrase occurring was “covid pandemic”, which appeared five times in the titles and 135 times in the abstracts. The most frequent three-word phrase was “post pandemic era”, occurring just twice in the 291 abstracts (see OSF supporting File J). We did not pursue further modifications to the draft search filters, as these phrases could not be excluded without adversely affecting recall.

We exceeded our target of 64% for precision in both databases and in the overall test of deduplicated records. We proceeded to validation without making further changes to the draft search filters.

### Validation

To validate the draft search filters, we downloaded records for the gold-standard set from the Cochrane COVID-19 Study Register on May 3, 2023. The Study Register contained 224,665 records in total, of which 28,884 were labelled as “Treatment and Management”, including 22,074 categorized as “Journal Articles”. We downloaded all 22,074 records in four batches. Once we had removed duplicates and trial registry entries, the master list contained 20,739 records.

We searched for these 20,739 records in MEDLINE, using PubMed ID where available, DOI number if known, or title. This identified 14,963 records in MEDLINE on May 19, 2023, which we downloaded in RIS files for further processing in EPPI-R5. We removed 142 duplicates and cleansed the data, removing 196 obviously irrelevant records, such as those with errors in the DOI field. The MEDLINE gold-standard set had 14,625 records, which we exported from EPPI-R5 and converted to Ovid format using the PubMed ID field (see OSF supporting File B).

We followed a similar process for Embase, where we searched for the 20,739 records using the accessionnumber where available, then the DOI number, followed by title, if neither of those were available. We had 20,239 results on May 19, 2023. We imported these records into EPPI-R5, removing 491 duplicates and 377 obviously irrelevant records. We obtained the Embase accession numbers for the remaining 19,371 records and created an Ovid strategy to retrieve them (see OSF supporting File C).

We ran the gold-standard sets and combined them with the draft search filters on May 19, 2023, using the Ovid segments dated May 18, 2023, in both databases. In MEDLINE, the recall was 99.86%, with the filter finding 14,604 and missing 21 of the 14,625 records in the gold-standard set (see Table 4). The Embase filter achieved 99.88% recall, finding 19,348 and missing 23 records. Both recall figures exceeded our target for performance.

**Table 4:** Relative recall when validating the filters (May 19, 2023).

Test set	MEDLINE	Embase
No. in gold-standard set	14625	19371
No. retrieved by filter	14604	19348
Percentage of those available retrieved by filter	99.86%	99.88%

The validated filters are presented in Figure 2 and are also available in OSF supporting File M to encourage reuse.

### Characteristics of the Missed Records

We found that the missed records would be of minimal importance to a literature search being conducted according to our definitions in Figure 1. The results are summarized in Table 5 (see OSF supporting Files K and L for the list of records and how we categorized them).

**Table 5:** Characteristics of the records from the gold-standard sets missed by the filters.

		MED LINE	Emb ase
No. of records missed from the gold-standard set		21	23
Format	Conference abstract	0	2
	Correction	13	3
	Journal article	6	10
	Journal article - case report	0	3
	Letter	2	5
Topic of the article	ARDS or mechanical ventilation	1	2
	COVID-19	16	13
	MIS or MIS-C	4	8
Title of the article	Refers to COVID-19 correctly in title (.ti)	1	0
	Error in reference to CO VID-19 in title (.ti)	1	1
	Refers to ARDS or dysfunction	0	2
	Refers to CO VID-19 in original title (.ot)	1	4
	Refers to MIS-C	3	8
	Refers to pandemic	1	2
	Contains no terms relating to a condition	14	5
	No title in the Ovid record	0	1
Abstract	No abstract	19	12
	Does not refer to COVID-19	2	2
	Refers to ARDS or dysfunction	0	1
	Refers to MIS-C	0	7
	Refers to pandemic	0	1
Keyword headings	Refers to COVID-19, SARS-CoV-2 or coronavirus	6	3
	Refers to ARDS or dysfunction	0	2
	Refers to MIS-C	0	3
	None referring to COVID-19	0	3
	None	15	12
Language	English	19	16
	French	0	2
	German	1	1
	Norwegian	0	1
	Spanish	1	2
	Swedish	0	1
	2019	1	0
Year of publication in Ovid record	2020	3	8
	2021	11	10
	2022	6	3
	2023	0	2

We found that corrections accounted for 13 records in MEDLINE, some of which only had the title “Erratum” (see Table 5). Five MEDLINE and 10 Embase records were about Multisystem Inflammatory Syndrome in Children (MIS-C) or Acute Respiratory Distress Syndrome (ARDS). One MEDLINE record had a spelling mistake in the title (“COVD-19”) and did not have an abstract or any MeSH terms [32]. The 10 Embase records about COVID-19 were difficult to find using free text, as one had no title, one had a mistake in the title (“theCOVID-19”), 12 had no abstract and four were records in other languages that used Original Title (.ot) instead of the Title field. We only missed one full journal article about COVID-19 in MEDLINE and that was because the Ovid record had a publication date of 2019, when it had been published online in January 2021 [33]. The one remaining journal article in English missed in Embase referred only to “a pandemic” [34].

## DISCUSSION

### Keeping the Search Filters up to Date

The filters incorporate a range of free-text terms and subject headings. We have only included free-text terms that add value to the filters. The filters retain some free-text terms (such as “2019nCoV” and “19nCoV”) that were used before the WHO naming conventions were more widely adopted. We have either removed or rejected a list of around 100 other words and phrases that would not improve recall, such as “SARS-CoV-2019” or “nCoV2019” (see the full list in Appendix B). The filters could miss some papers published in January 2020 that identified the initial outbreak in Wuhan, although it is unlikely these would refer to treating or managing COVID-19.

It is important to keep the filters up to date, testing new terminology (e.g. variants of interest or concern) and subject headings. The filters exceed our recall targets without having to include free-text terms referring to “Delta” or the earlier variants. We did have to include title and keyword searches for the term “Omicron” to maintain recall. The Keyword Heading Word field is useful because it is populated by the authors of the studies, who are likely to name a new variant before it has been included in MeSH or Emtree. In the Embase filter, we have exploded the subject heading “Severe acute respiratory syndrome coronavirus 2” to ensure it retrieves any new, as yet unnamed, variants as soon as they are added to Emtree. The MEDLINE filter is more stable, as the subject heading “SARS-CoV-2” is currently used for all variants without having any narrower headings.

The timing of any subsequent updates to the search filters is difficult to predict, as testing cannot take place as soon as WHO identifies a new variant of interest or concern. We need to wait until the variant is discussed in the literature and then keep the terms under review to assess the impact on recall. It also takes time for new subject headings to be added into MeSH and Emtree and for these to be made available in Ovid. We may need to expand the free text in the early stages after a new variant is identified, before making later versions of the filters more precise, once the subject headings have been updated.

### Coverage of Other Pandemics and Coronaviruses

It can be difficult to distinguish between articles that are about COVID-19 and those that are referring to events that occurred during the pandemic. We found that abstracts referring to events that happened “during the pandemic” were not usually about treating or managing COVID-19. Our filters do not cover general pandemic preparedness, as this may include other diseases, such as influenza. Our filters were already achieving their target for recall and so we did not alter them to retrieve more of these general “pandemic preparedness” records, which would have also reduced precision.

We are aware from the precision test (see Table 3) that the filters do retrieve records about other coronaviruses. We chose not to make the filters more precise as we did not want to exclude records comparing coronaviruses, such as a review of treatments for COVID-19, MERS and SARS. We also chose subject headings specific to COVID-19 and SARS-CoV-2 from lower in the MeSH and Emtree hierarchies to avoid retrieving records about feline coronavirus, porcine delta coronavirus or other coronaviruses outside of our definitions in Figure 1. We included a date limit in the filters to minimize the retrieval of records about other coronaviruses that were published before January 2020.

### Coverage of Conditions Secondary to COVID-19

We defined the parameters of the filters in Figure 1 to refer to the specific condition COVID-19. We chose not to expand the remit of the filters to cover conditions that are secondary to COVID-19, such as MIS-C, ARDS, cytokine storm or Kawasaki disease.

We felt that retrieving records on these secondary conditions could be done in one of two ways. Firstly, the filters already adequately retrieve records where the searcher is only interested in a condition when it is caused by COVID-19 (e.g. all records retrieved by “Cytokine storm AND COVID-19” would be found by the filters). Secondly, a comprehensive search for a disorder that can be triggered either by COVID-19 or another condition needs its own strategy and not a COVID-19 filter. For example, Vaccine Induced immune Thrombocytopenia and Thrombosis (VITT) ought to be searched in its own right, as it is not necessarily caused by a COVID-19 vaccine. Therefore, we did not expand our filters to increase recall of these secondary conditions.

Similarly, we decided that the filters should not cover post-COVID-19 syndrome (also known as long covid). We felt that searches to identify treatment and management strategies for this condition would need to be developed separately, rather than relying on a general COVID-19 filter.

### Measures to Increase Recall

We found that 15 of the 21 records we missed in MEDLINE during validation and 8 of the 23 in Embase were letters or corrections (see Table 5). There was often no way of recognizing that these were relevant from the Ovid records, without reviewing the full text. This suggests that after screening search results it is worth following up the potentially includable studies for related letters, corrections, retractions, editorials or comments [35]. A quick way to do this for COVID-19 studies is to use the Cochrane COVID-19 Study Register, which helpfully links together the references to a study in a single record [18].

We have designed the filters to balance our recall and precision targets. It would be possible to increase recall, at the expense of precision, by increasing the number of fields used for the free text. We missed a small number of records because their titles were in the Original Title (.ot) field. We could also retrieve some of the missed records by extending our use of keyword headings to other lines in the filters. The simplest way of making these changes would be to apply the Multi-Purpose field (.mp) to all of the free-text terms [6, 7].

We could also increase recall at the expense of precision by exploding more subject headings. We did not explode the Emtree term “Coronavirus disease 2019” in the Embase filter, as doing so would retrieve headings on a number of related conditions, including long covid, VITT and MIS-C. We have not tested how these changes affect precision, since our current filters exceed 99% recall.

## LIMITATIONS

We acknowledge that the search filters have been validated for use in searches requiring evidence on drugs, devices, surgical procedures and other therapeutics. We have not tested the recall of records about diagnosis, prognosis, transmission, prevention, vaccination, mechanisms of action, epidemiology or etiology. The filters are not suitable for searching for related conditions caused by COVID-19, as we have not included the subject headings required for these.

The first test set for recall was derived from another study but this had already been peer reviewed [24]. We also took steps to update the test set obtained from Butcher et al. to ensure coverage of later papers and variants of SARSCoV-2.

We were reliant on the Cochrane COVID-19 Study Register when creating our gold-standard set. As Cochrane compile the Study Register by searching several sources, we were testing our filters against a broad range of COVID-19 studies, rather than just comparing the filters to the search strategies that Cochrane use on individual databases. We also knew that the Study Register had 94.4% coverage of interventional studies in November 2020 [20]. We could rely on the papers in the Study Register being relevant to our gold-standard set because Cochrane assess their search results using a validated machine-learning classifier that has a recall rate of 98.9% [22]. The search and classification methods used for the Cochrane Study Register have been quality assured for maximum sensitivity of human studies and they are transparent, rigorous and high performing [20, 22]. We are confident that our gold-standard set accurately represents a sample of relevant literature on treating and managing COVID-19. We were able to obtain a much larger set than if we had hand searched for relevant records to include in the gold standard [5].

We noted in the discussion of the precision tests that papers referring to events that occurred “during the pandemic” will often refer to COVID-19 and be retrieved by these filters. The filters are only intended to retrieve records referring to people with diagnosed or suspected COVID-19.

## CONCLUSIONS

We have optimized the search filters for use in the Ovid versions of MEDLINE and Embase when needing to retrieve records about treating and managing COVID-19. We set targets of 98.9% for recall and 64% for precision. In the first recall test, both filters had 99.5% recall. In the second test, recall increased to 99.7% and 99.8% in MEDLINE and Embase respectively. The filters had a precision of 91.1% in a deduplicated sample of records. In validation, we found the MEDLINE filter had relative recall of 99.86% (finding 14,604 of the 14,625 records in the gold-standard set) and the Embase filter had 99.88% relative recall (finding 19,348 of 19,371 records). As with all search filters, there will be an ongoing need to keep them up to date by reviewing the free-text terms, subject headings and fields included. The validated search filters can be used in literature searches about treating and managing COVID-19.

## Data Availability

Data associated with this article are available in the Open Science Framework at https://osf.io/hwgke/.

## SUPPLEMENTAL FILES

**Appendix A:** Version history for the NICE search filters for treating and managing COVID-19

**Appendix B:** List of free-text terms considered during development of the filters

**Appendix C:** Description of the online-only supporting materials available from Open Science Framework (OSF)
