# Supplementary material for: The NICE search filters for treating and managing COVID-19: validation in MEDLINE and Embase (Ovid)
Source: J Med Libr Assoc. 2024 Jul 29;112(3):225–37. doi: 10.5195/jmla.2024.1806 (PMC11412126; doi:10.5195/jmla.2024.1806)
Supplement: Supplementary file 1 — Appendix A: Version history for the NICE search filters for treating and managing COVID-19 [file jmla-112-3-225-s01.pdf]

Appendix A Version history for the NICE search filters for treating and managing COVID-19

The full search strategies for each Ovid MEDLINE version are available in the online-only supporting materials, see File A posted to OSF.

Version	Date implemented	Actions
v1	March 16, 2020	Terms suggested by Public Health England (now the UK Health Security Agency) added to MEDLINE and Embase in Ovid.
v2	March 17, 2020	Quality Assurance of v1:<br>• Added the free-text term "epidemic".<br>• Added the free-text term "OR Huanan" alongside the term "Wuhan".<br>• Replaced AND with adj10 to increase specificity on the free-text line for "outbreak".<br>• Added a free-text line for the phrase "severe acute respiratory syndrome*".<br>• Added the Keyword Heading field to capture authors' keywords as this was a new area where the indexing was not up to date.
v3	March 18, 2020	Increased the precision as it was retrieving too many records on other epidemics in China (e.g. malaria).<br>Split the lines on ("respiratory symptom*" or "seafood market") from ("outbreak* or wildlife* or pandemic") so that the adjacency could be narrowed.
v4	March 19, 2020	This version was added to the Interim process manual published on the NICE website.<br>• Identified and added the Emtree heading "Coronavirinae".<br>• Added "Coronavirinae" to the free text.

		• Added "respiratory condition" to the free text and remodeled this line.<br>• Made changes to the way that "Wuhan" and "Huanan" were included as free-text terms.
v5	March 21, 2020	Identified an additional MeSH heading:<br>• Added Coronavirus Infections/
v6	March 25, 2020	Made the strategies more specific following feedback they were still over retrieving records about other epidemics in China:<br>• Changed the position of "Wuhan" in the strategy but also added "pneumonia" to the line on respiratory conditions.<br>• Added the abbreviation "HCoV" for human coronaviruses to the free text; this had a small impact as the relevant subject headings were already incorporated by exploding the ones higher in the hierarchy.<br>• Undertook extensive testing in MEDLINE on the Keyword Heading and Keyword Heading Word fields (the fields were different in Embase).<br>• Changed all MEDLINE free-text lines to use the fields (.ti,ab,kw,kf)
v7	April 8, 2020	No substantive changes but some free-text lines were separated to make the strategy easier to read.
v8	April 16, 2020	Added terms to the free text for consistency:<br>• nCoV19 or "nCoV-19"<br>• HCoV19 or "HCoV-19"
v9	June 3, 2020	Undertook an in-depth review of the free-text lines and the structure:

		• Reviewed the free-text terms used for "COVID-19" and "SARS-CoV-2", including how they were truncated.<br>• Removed some of the free-text terms that had zero hits.<br>• Checked all lines for consistency, adding some truncation.<br>• Tested and decided not to add some additional terms that had been identified, such as "Betacoronavirus".<br>• Changed the structure slightly to remove references on pneumonia in China not relevant to this pandemic.
v10	April 16, 2021	Undertook extensive testing of each free-text line.<br>Added the new MeSH and Emtree headings that were now available in Ovid.<br>Did a comparison to the COVID-19 Limit that Ovid built into MEDLINE.<br>Completely restructured the strategies to make them more concise.<br><br>The strategies, as published in the June 2021 preprint:<br>• were developed for the NICE remit on COVID-19.<br>• did not include the MeSH headings "exp COVID-19 Testing" or "COVID-19 Vaccines".<br>• included subject headings for "COVID-19" and "SARS-CoV-2" rather than exploding the terms from higher in the hierarchy, as in previous versions.<br>• did not aim to cover comprehensively other coronaviruses (such as MERS or SARS).<br>• included a publication date limit of 2019-current to restrict the number of records on other coronaviruses.

		• was not tested for retrieval of records about variations of SARS-CoV-2 (no missing records identified).<br>• used COVID*2 to retrieve "COVID-19" and "COVID19" while avoiding retrieving terms such as "Covidence", meaning a small number of records about COVID-19 that use unusual terminology (e.g. "covidology") were not retrieved, although they were all verified as being out of the NICE remit.<br>• removed some of the free-text lines relating to "Wuhan" and "food markets", which were not relevant to managing or treating COVID-19 but were required to identify papers written in January 2020 about the early identification of the pandemic.<br>• did not include terms for related conditions that are not exclusively caused by COVID-19 e.g. Macrophage Activation Syndrome, Cytokine Release Syndrome and Multisystem Inflammatory Syndrome.<br>• incorporated a Boolean NOT to exclude some phrases (e.g. coefficients of variation) that are abbreviated to CoV but are not relevant to COVID-19.<br>• did not aim to cover general pandemic preparedness.<br>• was not optimized for long covid.
v11	November 16, 2021	Further testing and revisions were made:<br>• Testing showed that the date limit could be changed from 2019 to 2020 to make the strategy more precise.<br>• Free-text terms for "anticovid" tested and rejected.<br>• Free-text terms for the Delta variant tested and rejected as they did not improve recall of records relevant to NICE.<br>• The appropriate updates were completed as Ovid updated Embase in September 2021 so that it has the

		same Keyword Heading and Keyword Heading Word fields as MEDLINE.
v12	April 24, 2023	Further testing and revisions were made before validation with the gold-standard set.<br>• Omicron.ti,kf was added to improve recall.<br>• More extensive terms for Variants of Interest or Concern were tested and rejected as they did not improve recall.<br>• The MeSH headings COVID-19 Drug Treatment/ and COVID-19 Serotherapy/ were tested and added to the MEDLINE search strategy.<br>• Tested and decided not to explode the Emtree heading "Coronavirus Disease 2019" to avoid adversely affecting precision with recall of records about secondary conditions, such as VITT.
Draft search filters	April 24, 2023	Adopted version 12 as the draft search filters for testing and validation in this paper.
