# Supplementary material for: The NICE search filters for treating and managing COVID-19: validation in MEDLINE and Embase (Ovid)
Source: J Med Libr Assoc. 2024 Jul 29;112(3):225–37. doi: 10.5195/jmla.2024.1806 (PMC11412126; doi:10.5195/jmla.2024.1806)
Supplement: Supplementary file 2 — Appendix B: List of free-text terms considered during development of the filters [file jmla-112-3-225-s02.pdf]

Appendix B List of free-text terms considered during development of the filters

Terms with the status Removed were in earlier versions (v1 to v11) before validation of the filters. Terms with the status Rejected have never been required to maintain recall performance. Further details are available in in the online-only supporting materials, see File D posted to OSF.

Free-text terms considered	Status in the search filters
"2019-nCoV"	Removed
"2019-nCoV*"	Removed
"CORVID-19"	Removed
"CoV 19*"	Rejected
"CoV 2*"	Rejected
"COVID-19"	Removed
"HCoV-19"	Removed
"HCoV-2019"	Removed
"nCoV-19"	Removed
"nCoV-19*"	Removed
"nCoV-2019"	Removed
"nCoV-2019*"	Removed
"SARS coronavirus2"	Removed
"SARS coronovirus2"	Removed
"SARS-2"	Removed
"SARScoronavirus 2"	Removed
"SARS-coronavirus-2"	Removed
"SARScoronovirus 2"	Removed
"SARS-coronovirus-2"	Removed

"SARSCov-19"	Removed
"SARS-Cov19"	Removed
"SARS-Cov-19"	Removed
"SARSCoV-2"	Removed
"SARS-CoV2"	Removed
"SARS-CoV-2"	Removed
"SARSCovID-19*"	Rejected
"SARS-CovID19*"	Rejected
"SARS-CovID-19*"	Rejected
"SARSCoVID2*"	Rejected
"SARS-CoVID2*"	Rejected
"SARSCovID-2019*"	Rejected
"SARS-CovID2019*"	Rejected
"SARS-CovID-2019*"	Rejected
"WN-CoV"	Removed
"wuhan virus*"	Rejected
(("respiratory illness*" or "respiratory disease*" or "respiratory symptom*" or "seafood market*" or "food market*") adj10 (Wuhan* or Hubei* or China* or Chinese* or Huanan*))	Removed
(("seafood market*" or "food market*" or pneumonia*) adj10 (Wuhan* or Hubei* or China* or Chinese* or Huanan*))	Removed
(((respiratory* adj2 (symptom* or disease* or illness* or condition*)) or "seafood market*" or "food market*" or pneumonia*) adj10 (Wuhan* or Hubei* or China* or Chinese* or Huanan*))	Removed
(((respiratory* adj2 (symptom* or disease* or illness* or condition*)) or "seafood market*" or "food market*") adj10 (Wuhan* or Hubei* or China* or Chinese* or Huanan*))	Removed

((alpha* or beta* or gamma* or delta* or lambda* or mu or "B.1.1.7" or "B 1 1 7" or B117 or "B.1.351" or "B 1 351" or B1351 or "P.1" or "P 1" or P1 or "B.1.617.2" or "B 1 617 2" or B16172 or "C.37" or "C 37" or C37 or "B.1.621" or "B 1 621" or B1621) adj (variant* or variation* or VOC or VOCs or VOI or VOIs or VOCI or lineage* or strain*)).ti,ab,kw,kf.	Rejected
((corona* or corono*) adj1 (virus* or viral* or virinae*))	Removed
((corona* or corono*) adj1 (virus* or viral*))	Removed
((outbreak* or "respiratory illness*" or "respiratory disease*" or "respiratory symptom*" or "seafood market*" or "food market*" or wildlife* or pandemic* or epidemic*) adj10 (Wuhan* or Hubei* or China* or Chinese* or Huanan*))	Removed
((outbreak* or "respiratory illness*" or "respiratory disease*" or "respiratory symptom*" or "seafood market*" or "food market*" or wildlife*) adj10 (Wuhan* or Hubei* or China* or Chinese* or Huanan*))	Removed
((outbreak* or wildlife* or pandemic* or epidemic*) adj1 (China* or Chinese* or Huanan*))	Removed
((outbreak* or wildlife* or pandemic* or epidemic*) adj1 (Wuhan* or Hubei* or China* or Chinese* or Huanan*))	Removed
((outbreak* or wildlife* or pandemic* or epidemic*) adj1 (Wuhan* or Hubei* or China* or Chinese* or Huanan*))	Removed
(Omicron* or "B.1.1.529" or "B1.1.529" or B11529 or "BA.5" or BA5 or "BF.7" or BF7 or "BF.14" or BF14 or "BQ.1" or BQ1 or "BQ.1.1" or "BQ1.1" or BQ11 or "BA.2.75" or "BA2.75" or BA275 or "BA.2" or BA2 or "BA.4.6" or "BA4.6" or "BA46" or "BA.4" or BA4 or XBB or "XBB.1.5" or "XBB1.5" or XBB15 or "BA.2.3.20" or "BA2.3.20" or BA2320 or "BA.2" or BA2 or "BA.4/5" or "BA4/5" or "BA.4.5" or "BA4.5" or BA45 or XBC or "BN.1" or BN1 or "CH.1.1" or CH11 or "CH1.1" or "CH.1.1.1" or "CH1.1.1" or CH111 or "CH.1.1.2" or "CH1.1.2" or CH112 or XBF)	Rejected
(pneumonia* adj3 (Wuhan* or Hubei* or China* or Chinese* or Huanan*))	Removed

(respiratory* adj2 (symptom* or disease* or illness* or condition*) adj10 (Wuhan* or Hubei* or China* or Chinese* or Huanan*))	Removed
(respiratory* adj2 (symptom* or disease* or illness* or condition*) adj5 (Wuhan* or Hubei* or China* or Chinese* or Huanan*))	Removed
2019nCoV	Removed
anticorona*	Rejected
anticov*	Rejected
antincov*	Rejected
anti-sars*	Rejected
Betacoronavirus*	Rejected
Betacoronovirus*	Rejected
coronavirinae*	Removed
coronovirus*	Removed
CORVID19	Removed
CoV	Removed
COV19*	Rejected
CoV2*	Rejected
COVID19	Removed
HCoV19	Removed
HCoV2019	Removed
Huanan	Removed
Hubei*	Removed
Ncorona*	Removed
Ncorono*	Removed

Ncov	Removed
nCoV19	Removed
nCoV19*	Removed
nCoV2019	Removed
nCoV2019*	Removed
NcovChina*	Removed
NcovChinese*	Removed
NcovHubei*	Removed
Ncovor	Removed
NcovWuhan*	Removed
Omicron*	Rejected
SARS 2 coronavirus*	Rejected
SARS2	Removed
SARS-2-Cov	Rejected
SARS-2-nCoV	Rejected
SARScoronavirus2	Removed
SARScoronovirus2	Removed
SARSCov19	Removed
SARSCoV2	Removed
SARSCovID19*	Rejected
SARSCovID2019*	Rejected
WNCoV	Removed

Wuhan*	Removed
