# Supplementary material for: The NICE search filters for treating and managing COVID-19: validation in MEDLINE and Embase (Ovid)
Source: J Med Libr Assoc. 2024 Jul 29;112(3):225–37. doi: 10.5195/jmla.2024.1806 (PMC11412126; doi:10.5195/jmla.2024.1806)
Supplement: Supplementary file 3 — Appendix C: Description of the online-only supporting materials available from Open Science Framework (OSF) [file jmla-112-3-225-s03.pdf]

Appendix C Description of the online-only supporting materials available from Open Science Framework (OSF)

All of the supporting online-only files associated with this article are available in the Open Science Framework at https://osf.io/hwgke/.

File	Format	Title	Contents
File A	Microsoft Word	Version history for Ovid MEDLINE	Includes the MEDLINE search strategies for all 12 versions
File B	Microsoft Word	Search strategies for testing and validation in Ovid MEDLINE	Includes the strategies for MEDLINE for Recall Test 1, Recall Test 2, Precision Test and Validation
File C	Microsoft Word	Search strategies for testing and validation in Ovid Embase	Includes the strategies for Embase for Recall Test 1, Recall Test 2, Precision Test and Validation
File D	Microsoft Excel	List of free-text terms considered	List of terms considered during development of the filters
File E	Microsoft Excel	Recall test 1: set obtained from Butcher et al. (2022)	Includes the list received from Butcher et al. and the updated data used in this paper
File F	Microsoft Excel	Recall test 2: supplementary test set	Includes the list downloaded from Epistemonikos, the relevant systematic reviews and the list of primary studies obtained from citationchaser
File G	Microsoft Excel	Precision test: screening decisions for MEDLINE	Lists showing the screening decisions made for the

			records obtained from Ovid MEDLINE
File H	Microsoft Excel	Precision test: screening decisions for Embase	Lists showing the screening decisions made for the records obtained from Ovid Embase
File I	Microsoft Excel	Precision test: screening decisions for deduplicated records	Lists showing the screening decisions made for the deduplicated test set
File J	Microsoft Excel	Precision test: frequency counts	Frequency counts obtained from <www.rapidtables.com> for the items excluded in the precision tests
File K	Microsoft Excel	Gold-standard set for Ovid MEDLINE	Includes the gold-standard set downloaded from the Cochrane COVID-19 Study Register and cleaned for validation in MEDLINE
File L	Microsoft Excel	Gold-standard set for Ovid Embase	Includes the gold-standard set downloaded from the Cochrane COVID-19 Study Register and cleaned for validation in Embase
File M	Microsoft Word	NICE COVID-19 search filters	A downloadable file containing the search filters to encourage their reuse
